# Changes in plasma arylsulfatase A level as a compensatory biomarker of early Parkinson’s disease

**DOI:** 10.1038/s41598-020-62536-4

**Published:** 2020-03-27

**Authors:** Han Soo Yoo, Jun Sung Lee, Seok Jong Chung, Byoung Seok Ye, Young H. Sohn, Seung-Jae Lee, Phil Hyu Lee

**Affiliations:** 1 Department of Neurology, Yonsei University College of Medicine, Seoul, South Korea; 2 Department of Biomedical Sciences, Neuroscience Research Institute, Seoul National University College of Medicine, Seoul, Korea; 3 Severance Biomedical Science Institute, Yonsei University College of Medicine, Seoul, South Korea

**Keywords:** Parkinson's disease, Neurological disorders

## Abstract

Lysosomal dysfunction has been associated with Parkinson’s disease (PD). However, the activity of lysosomal enzymes is heterogeneously observed in PD. We investigated whether arylsulfatase A (ARSA) level can be used as a fluid biomarker of PD and can reflect disease progression. Plasma ARSA level was measured in 55 patients with early and drug-naïve PD, 13 patients with late PD, and 14 healthy controls. We compared the plasma ARSA level among the groups and assessed its correlation to clinical parameters and striatal dopamine transporter (DAT) activity. Plasma ARSA level was not correlated with age. The early PD group had higher plasma ARSA level than the control and late PD groups. In a generalized additive model including all patients with PD, the plasma ARSA level showed an inverted U-shape according to disease duration, peaking at 2.19 years. In patients with early PD, plasma ARSA level was positively correlated to parkinsonian motor score and negatively to striatal DAT activity. In summary, plasma ARSA level was elevated in early stage of PD, and elevated plasma ARSA level was correlated to the clinical and imaging markers of nigrostriatal degeneration. These results suggest that ARSA level is a potential biomarker of compensation in early PD.

## Introduction

In about 5–10% of patients, Parkinson’s disease (PD) is caused by rare, inherited forms of genetic mutations^1^, and a genome-wide association study has identified 24 loci that are linked to an altered risk of developing PD^2^. Representatively, *GBA* gene, the causative gene for Gaucher’s disease, which is the most common lysosomal storage disease^3^, also constitutes the single largest risk factor of idiopathic PD^4,5^. Considering that many genes known as risk factors for PD are relevant for intracellular transport pathway to lysosomes or lysosomal function^6^, defects in this system and consequent accumulation of aggregated protein play a primary role in the pathogenesis of PD. Accordingly, studies have identified the fluid biomarker of lysosomal enzymes to measure the central pathological process in PD^7–9^. In particular, patients with PD have reduced level of glucocerebrosidase in the blood and cerebrospinal fluid (CSF), which provides a value in diagnosing PD^7,10^.

Arylsulfatase A (ARSA) is a lysosomal acid hydrolase, whose deficiency causes metachromatic leukodystrophy, an autosomal recessive lysosomal storage disease^11^. A recent study has revealed that *ARSA* gene mutations acted as a genetic modifier, either pathogenic or protective, in PD^12^. Specifically, *ARSA* gene deficiency facilitated α-synuclein aggregation and cell-to-cell propagation through the molecular chaperone function of ARSA. Lower ARSA level has also been shown to be correlated with cognitive dysfunction.

In this study, we examined the plasma ARSA level and its associations with clinical and imaging parameters of PD in order to elucidate whether ARSA can be used as a fluid biomarker of PD and can reflect disease progression.

## Results

The baseline demographic and clinical characteristics of the patients are summarized in Table 1. The early PD group had younger age at the time blood sampling than the late PD group, whereas the ages of the two groups were comparable with the healthy controls (HC) group. The PD groups had lower total K-MMSE scores than the HC group, and the late PD group had lower total K-MMSE scores than the early PD group. The sex ratio and years of education were not different among the groups. Between the PD groups, the late PD group had longer disease duration and higher UPDRS motor score than the early PD group, whereas both groups developed PD at similar ages.

**Table 1:** Demographic and clinical characteristics of patients with healthy control and Parkinson’s disease.

Variables	HC	Early PD group	Late PD group	*P*-value
Number	14	55	13	
Age at blood sampling, y	72.5 (66.1–76.3)	69.8 (62.4–75.1)	74.6 (70.7–78.9)	0.047^c^
Sex, male, *n* (%)	5 (35.7)	30 (54.5)	7 (53.8)	0.443
Education, y	12.0 (11.3–16.5)	12.0 (6.0–14.0)	9.0 (3.5–13.0)	0.081
Total K-MMSE score	28.5 (28.0–30.0)	27.0 (26.0–28.0)	22.0 (19.0–24.5)	<0.001^a,b,c^
Age at PD onset, y		67.1 (61.3–73.6)	65.4 (60.1–70.3)	0.507
Disease duration, y		1.5 (0.6–2.2)	8.1 (7.4–11.5)	<0.001
UPDRS motor score (off)		19.0 (14.0–27.0)	45.0 (33.5–52.0)	<0.001
RBD, *n* (%)		22 (40.0)	10 (76.9)	0.016
Medication duration, y			7.5 (5.6–10.6)	NA

Values are expressed as median (interquartile range) or number (percentage).

Significant difference between ^a^HC vs. early PD, ^b^HC vs. advanced PD, ^c^early PD vs. advanced PD after host-hoc group comparison of Bonferroni procedure.

Abbreviations: K-MMSE, the Korean version of the Mini-Mental State Examination; NA, not applicable; PD, Parkinson’s disease; RBD, rapid eye movement sleep behavior disorder; UPDRS, Unified Parkinson’s Disease Rating Scale.

**Figure 1:**
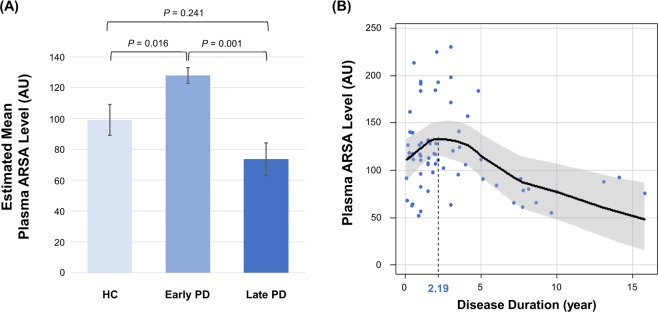
(**A**) Group comparison of plasma ARSA levels. The post-hoc subgroup comparison was performed using the Bonferroni method. (**B**) Relationship between plasma ARSA level and disease duration. In all analyses, age, sex, and total K-MMSE score were used as covariates. ARSA, arylsulfatase A; AU, arbitrary unit; HC, healthy controls; K-MMSE, Korean version of the Mini-Mental State Examination; PD, Parkinson’s disease.

Independent of age, sex, and total K-MMSE score, the early PD group had significantly higher estimated mean plasma ARSA level than the HC group (127.87 vs. 99.10, *p* = 0.016) and late PD group (127.87 vs. 73.62, *p* = 0.001), whereas that of the late PD group had a trend to be lower than that in the HC group (*p* = 0.080, Fig. 1A). In a generalized additive model including all patients with PD, the plasma ARSA level showed an inverted U-shape according to disease duration, with a peak at 2.19 years (Fig. 1B).

**Figure 2:**
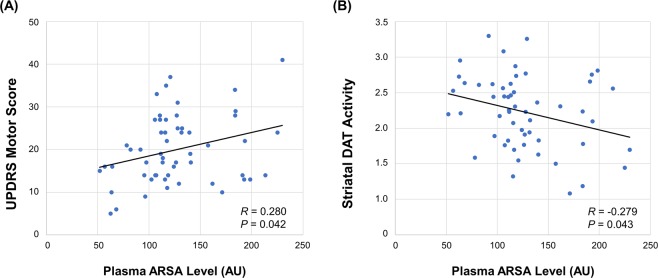
(**A**) Correlation between plasma ARSA level and the UPDRS motor score. (**B**) Correlation between plasma ARSA level and the striatal dopamine transporter activity. In all analyses, age, sex, and total K-MMSE score were used as covariates. ARSA, arylsulfatase A; AU, arbitrary unit; DAT, dopamine transporter; K-MMSE, Korean version of the Mini-Mental State Examination; PD, Parkinson’s disease; UPDRS, Unified Parkinson’s Disease Rating Scale.

In the early PD group, the plasma ARSA level was positively correlated to UPDRS motor score (*r* = 0.280, Fig. 2A) and negatively correlated to striatal DAT activity (*r* = −0.279, Fig. 2B). Other clinical parameters, such as age at onset, age at sampling, years of education, and total K-MMSE score, were not correlated to plasma ARSA level in patients with early or late PD.

## Discussion

The present study showed that the level of plasma ARSA had an inverted U-shape according to disease duration, with a peak at about 2 years. In the early stage of PD, the plasma ARSA level was positively correlated to UPDRS motor score and striatal dopamine depletion, thereby reflecting the disease severity of PD. Considering that ARSA deficiency has been shown to be associated with an increase in α-synuclein aggregates^12^, these data indicated that the plasma level of ARSA, a lysosomal enzyme, is elevated in the early stage of PD, which could be a novel biomarker of the compensation of nigrostriatal degeneration.

Several studies have shown a heterogeneous level of lysosomal enzyme activities in fluid biomarker or brain tissue in patients with PD. Glucocerebrosidase activity is consistently lower in the blood^13^, CSF^7^, or brain tissue^14^ in patients with PD than in normal controls, whereas the activities of serum and CSF β-galactosidase or CSF β-hexosaminidase are increased^8,9,15^. Considering that varying levels of lysosomal enzyme activity were found in patients with PD with a wide range of disease duration, dynamic change in the activity of each lysosomal enzyme as PD progresses must be investigated. In this study, we showed a different pattern of ARSA activity according to PD duration, indicating that plasma ARSA levels increased within the first 2 years of the disease and then decreased as the disease progressed. In addition, the activity of ARSA in the early stage of PD well correlated to the clinical and imaging parameters of degeneration in the nigrostriatal dopaminergic neuron. Accordingly, the higher activity of ARSA in the early stage is either a direct marker of lysosomal dysfunction or a compensation of autophagic clearance of aggregated α-synuclein in neurons. Considering that ARSA depletion promoted intracellular accumulation and intercellular propagation of α-synuclein and that lower ARSA level was correlated with cognitive dysfunction in our previous study^12^, the PD duration-specific pattern of ARSA activity in the current study indicated that deficiency in ARSA activity may lead to detrimental effects on nigrostriatal dopaminergic neurons. Thus, elevation of ARSA activity in relation to nigrostriatal degeneration in the early stage of PD might be a compensatory phenomenon in response to intracellular accumulation of aggregated α-synuclein. This result would provide a new perspective on the role of lysosomal enzyme in the early stage of PD and its importance as a fluid biomarker of disease progression.

Although the exact source and tissue contribution of plasma ARSA have not been fully elucidated, ARSA is broadly expressed in brain residing cells and in lymphocytic cells in the blood, spinal cord, and peripheral tissues, and is also secreted from cells^16^. In addition, a previous study has reported that some exosomes present in the sera contain lysosomal enzymes^17^. In Alzheimer’s disease, the levels of plasma lysosomal enzymes vary depending on disease progression and are more sensitive to cellular metabolic alteration compared to the levels of amyloid-β peptide or tau proteins^18^. Moreover, when considering the peripheral-onset hypothesis of the formation of abnormal α-synuclein aggregates^19^, alteration in the activity of lysosomal enzyme in peripheral blood may already reflect the general lysosomal dysfunction in PD. As in the case of other lysosomal enzymes, our data provide further evidence showing that plasma ARSA is correlated to PD progression. Future studies on whether the level of lysosomal enzyme in the peripheral blood is correlated to the activity of lysosomal enzyme in brain samples with PD or dopaminergic neurons are needed.

The present study has several limitations: First, this is a cross-sectional study and, therefore, provides limited grounds for a compensatory role of ARSA in individual level, longitudinally. Serial measures of the plasma ARSA level are required to elucidate actual changes in the plasma ARSA as PD progresses. Second, this study was based on a relatively small sample, especially the control and late PD groups, which limits the generalizability of our study. Third, we quantified the plasma ARSA level using Western blot. Since we were unable to measure ARSA level in human plasma samples using ARSA activity assay, it is necessary to develop a more accurate method for measuring ARSA levels in plasma and demonstrate its validity and reliability as a compensatory biomarker for early PD.

In summary, the plasma ARSA level was elevated in the early stage of PD in relation to nigrostriatal degeneration and was reduced in the late stage, showing an inverted U-shape according to disease duration. These results indicated that the peripheral metabolism of lysosomal enzymes could be a potential biomarker of compensation in early PD.

## Materials and Methods

### Participants

We consecutively recruited HC and patients with PD between June 2015 and November 2017 at the movement disorders outpatient clinic in Yonsei University Health System. The participants in the control group did not have any subjective symptoms of cognitive impairment or a history of neurologic or psychiatric illnesses. All participants in the control group had normal cognitive function according to Korean version of the Mini-Mental State Examination (K-MMSE scores ≥ 26). We have recruited two groups of patients with PD: one group had less than 5 years of disease duration with drug-naïve state (early PD group, n = 55) and the other group had more than 5 years of disease duration and were taking dopamine medications (late PD group, n = 13). Diagnosis of PD was based on the clinical diagnostic criteria of the United Kingdom PD Society Brain Bank^20^, and only patients diagnosed with PD who responded to dopaminergic medication during the follow-up period (≥ 6 months) were included in this study. The early PD group in this study had undergone *N*-(3-[^18^F]fluoropropyl)−2β-carbon ethoxy-3β-(4-iodophenyl) nortropane (^18^F-FP-CIT) positron emission tomography (PET) imaging at the time of their PD diagnosis, which revealed decreased uptake. The exclusion criteria included any evidence of motor, autonomic, oculomotor, and cognitive or neurobehavioral features indicative of atypical parkinsonism^21^; focal brain lesions, severe white matter hyperintensities^22^, multiple lacunes in the basal ganglia, or hydrocephalus on magnetic resonance imaging; or other neurologic, psychiatric, or metabolic illnesses. Parkinsonian motor symptoms were assessed during the drug-naïve state at the time of ^18^F-FP-CIT PET acquisition using the Unified PD Rating Scale motor (UPDRS) part III subscales. The K-MMSE was used to assess general cognition.

This study was approved by the Institutional Review Board of Yonsei University Severance Hospital (4-2013-0407). A written informed consent was obtained from all patients and healthy controls who participated in this study. All experiments were performed in accordance with the approved guidelines of the Institutional Ethical Committee.

### Human plasma preparation

In total, 10 mL of plasma samples were drawn via venous puncture and were placed into EDTA tubes. Immediately after filling each tube, it was gently inverted 10 times. The blood tubes were allowed to stand at room temperature for approximately 30 min prior to centrifugation, which was performed for 20 mins at 2000 rpm at 4 °C. The supernatant was aliquoted into 500 cc in 1.5 mL cryogenic vials. The aliquot of plasma was frozen in a freezer with a temperature of −75 °C or −80 °C until use.

### Western blot analysis of plasma ARSA levels

**Figure 3:**
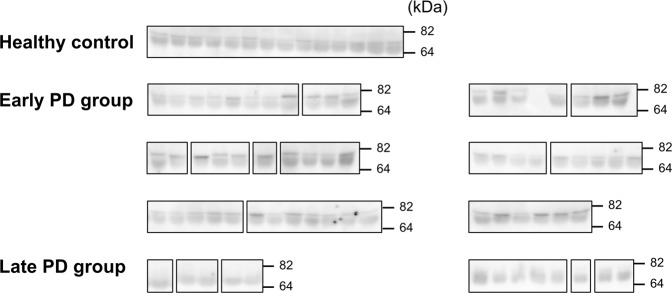
Western blot images (cropped) of plasma ARSA levels. Western blot analysis of plasma ARSA levels was performed in healthy controls (N = 14), early Parkinson’s disease (PD) group (N = 55), and late PD group (N = 13).

We measured the plasma ARSA level of the study participants using Western blot analysis (cropped images in Fig. 3; uncropped images in Supplementary Figure S1). To analyze ARSA protein levels, plasma preparations were mixed with SDS sample buffer and heated at 95 °C for 5 min. The samples were dissolved by 12% SDS-PAGE gel and then transferred onto a nitrocellulose membrane. After incubation with PBS containing 0.1% Tween-20 and 5% skim milk for 30 min, the membrane was probed with anti-ARSA antibody (1:1,000; Abcam, ab174844, Cambridge, MA), followed by HRP-conjugated goat anti-rabbit IgG (1:5,000; Jackson Immunoresearch Laboratories, West Grove, PA). The chemiluminescence detection was performed using the Amersham Imager 600 (Ge Healthcare Life Sciences, Marlborough, MA) and the band intensity was quantified using the NIH ImageJ software.

### PET-CT image acquisition

All participants were injected with 185 MBq (5 mCi) of 18F-FP-CIT and the PET images were obtained 90 min after injection. ^18^F-FP-CIT PET-CT acquisition was performed with a GE Discovery 600 PET/CT scanner (General Electric Healthcare, Milwaukee, MI, USA). The images were acquired for 15 min after CT scan for attenuation correction. The spiral CT scan was performed with the following parameters: 0.8 s/rotation at 120 kVp, 10 mA, 3.75-mm slice thickness, 0.625-mm collimation and 9.375-mm table feed per rotation. ^18^F-FP-CIT PET images were reconstructed using the ordered subset expectation maximization (OSEM) algorithm with four iterations and 32 subsets. Gaussian filter with 4-mm full-width at half-maximum (FWHM) was applied into the reconstructed PET images, which is a 256 × 256 matrix with 0.98-mm pixel and 0.98-mm slice thickness.

### Quantitation of the 18F-FP-CIT PET-CT images

Image processing was performed using the MATLAB (The MathWorks, Inc, Natick, MA) based software statistical parametric mapping (SPM8) and ITK-SNAP (http://www.itksnap.org). All reconstructed ^18^F-FP-CIT images were normalized to the ^18^F-FP-CIT template, which was made using the ^18^F-FP-CIT PET images and T1-weighted MRI images of the 40 healthy controls as described previously^23^. All healthy controls had no previous history of neurologic or psychiatric illness. They showed normal cognitive function based on all neuropsychological tests, and their neurologic examination, structural MRI, and ^18^F-FP-CIT PET findings were normal. For non-specific binding, the mean standardized uptake value was calculated by drawing two occipital volume-of-interests (VOI), one in each side. The level of DAT activity in striatal VOI was calculated in terms of the specific to non-specific binding ratio as follows: (mean standardized uptake of the striatal VOI – mean standardized uptake of the occipital VOI)/(mean standardized uptake of the occipital VOI).

### Statistical analyses

The baseline demographic and clinical characteristics of the patients with PD and normal controls were analyzed. We used the Mann–Whitney U test or Kruskal–Wallis test for continuous variables and Pearson’s χ^2^ test for categorical variables. Analysis of covariance was used to compare the plasma ARSA level among the groups. Post-hoc subgroup comparisons were performed using the Bonferroni method. The generalized additive model was used to investigate the non-linear relationship between the plasma ARSA level and disease duration in all patients with PD. Partial correlation analysis was performed to evaluate the association between the plasma ARSA level and UPDRS motor score or striatal DAT activity in patients with early PD. We adjusted for age, sex, and total K-MMSE score as covariates in all statistical analyses except for the comparison of baseline demographic and clinical characteristics. Data were analyzed using R v3.5.2 (Institute for Statistics and Mathematics, Vienna, Austria; www.r-project.org). *P*-values < 0.05 were considered significant.

## Supplementary information

Supplementary information.

## Data Availability

Data generated in the present study are available from the corresponding author upon reasonable request.
