# Supplementary material for: Changes in plasma arylsulfatase A level as a compensatory biomarker of early Parkinson’s disease
Source: Sci Rep. 2020 Mar 27;10:5567. doi: 10.1038/s41598-020-62536-4 (PMC7101326; doi:10.1038/s41598-020-62536-4)
Supplement: Supplementary file 1 — Supplementary information. [file 41598_2020_62536_MOESM1_ESM.pdf]

Changes in plasma arylsulfatase A level as a compensatory biomarker of early Parkinson's disease

Han Soo Yoo, MD[1], Jun Sung Lee[2], Seok Jong Chung, MD[1], Byoung Seok Ye, MD, PhD[1], Young H. Sohn, MD, PhD[1], Seung-Jae Lee, PhD[2], Phil Hyu Lee, MD, PhD[1,3*]

[1]Department of Neurology, Yonsei University College of Medicine, Seoul, South Korea

[2]Department of Biomedical Sciences, Neuroscience Research Institute, Seoul National University College of Medicine, Seoul, Korea

[3]Severance Biomedical Science Institute, Yonsei University College of Medicine, Seoul, South Korea

Corresponding author*: Phil Hyu Lee, MD, PhD

Department of Neurology, Yonsei University College of Medicine

50 Yonsei-ro, Seodaemun-gu, Seoul, 03722, South Korea

Tel.: 82-2-2228-1608

Fax.: 82-2-393-0705

E-mail: phlee@yuhs.ac

Supplementary Figure

S1. Uncropped Western blot images of plasma ARSA levels.

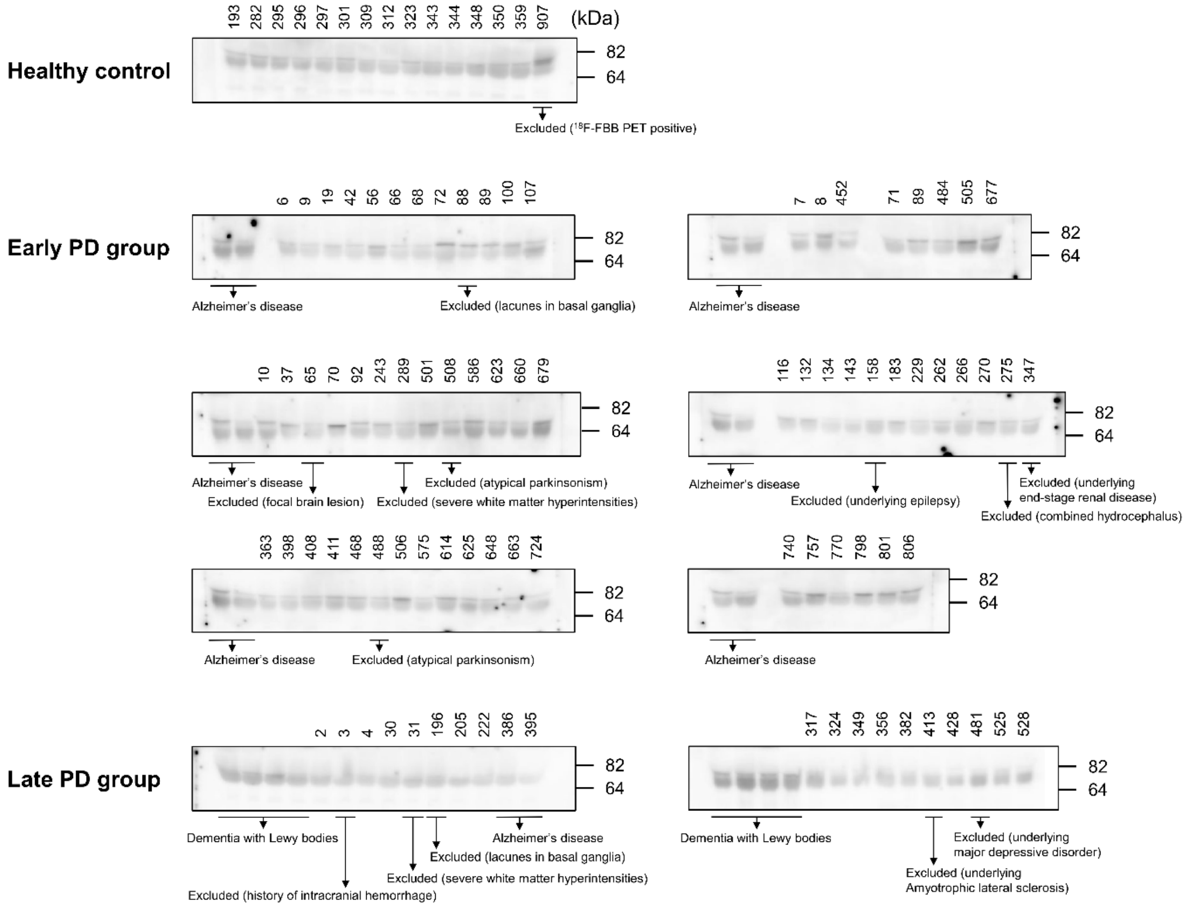

Western blot analysis of plasma ARSA levels was performed in healthy control, early Parkinson's disease (PD) group, and late PD group. One subject was excluded from the healthy control group, seven from the early PD group, and five from the late PD group. The number above the gel is the registration number of the blood sampling project (IRB No.: 4-2013-0407).
